# Fast and accurate Monte Carlo sampling of first-passage times from Wiener diffusion models

**DOI:** 10.1038/srep20490

**Published:** 2016-02-11

**Authors:** Jan Drugowitsch

**Affiliations:** 1 University of Geneva, Department of Basic Neuroscience, 1211 Geneva, Switzerland

## Abstract

We present a new, fast approach for drawing boundary crossing samples from Wiener diffusion models. Diffusion models are widely applied to model choices and reaction times in two-choice decisions. Samples from these models can be used to simulate the choices and reaction times they predict. These samples, in turn, can be utilized to adjust the models’ parameters to match observed behavior from humans and other animals. Usually, such samples are drawn by simulating a stochastic differential equation in discrete time steps, which is slow and leads to biases in the reaction time estimates. Our method, instead, facilitates known expressions for first-passage time densities, which results in unbiased, exact samples and a hundred to thousand-fold speed increase in typical situations. In its most basic form it is restricted to diffusion models with symmetric boundaries and non-leaky accumulation, but our approach can be extended to also handle asymmetric boundaries or to approximate leaky accumulation.

For a wide range of problems, human and animal decision-makers are known to trade-off the accuracy of choices with the speed with which these choices are performed. The dominant set of models to explain this trade-off for two-choice decisions is the diffusion model^1^,^2^,^3^,^4^. These models have been successfully applied to a wide range of data, from memory recall^1^ over lexical decisions^5^ to perceptual decisions^2^,^6^. Recently, the same set of models were shown to also account very well for the neural correlates associated with these decisions [e.g.^4^,^7^,^8^,^9^]. Thus, they have become a cornerstone of decision-making research.

In its simplest form, a diffusion model is formed by a particle whose trajectory follows a stochastic Wiener process with overlayed deterministic drift until one of two boundaries is reached (Fig. 1(a)). Each boundary triggers a different choice, and the time at which the particle first reaches this boundary is the first-passage time. Thus, each realization of such particle trajectory yields both a choice, and a time at which this choice was performed. The stochastic diffusion causes these times and choices to vary across different particle trajectory realizations. Thus, for the same drift and boundary locations, the model predicts a certain probability of performing either choice, each of which is associated with a distribution of first-passage times. These, in turn, can be compared to the behavior of humans and animals, and can be used to tune model parameters to match this behavior.

Choice probabilities and first-passage time densities can be computed either analytically, where such analytical expressions are known [e.g.^1^,^10^,^11^], or by repeatedly sampling choices and first-passage times by Monte Carlo simulations^12^. The sampling approach has several advantages. First, a diffusion model is easily sampled from using the Euler-Maruyama method [e.g.^13^], the stochastic extension of the Euler method, by simulating a particle trajectory in small time-steps. Second, such sampling is even possible for more complex variants of diffusion models, for which no analytical expressions are known. Third, even if such expressions exists, the sampling approach is usually easier to implement^12^. For example, even for the simple diffusion model described above the analytical expressions for the first-passage time densities involve infinite series that in practice need to be truncated and might be error-prone to implement. For such models, simple expressions for the mean first-passage time are known, but they are of limited utility as the full densities are heavily skewed. Fourth, simulating a diffusion model is most likely the easiest way to generate example behavior predicted by this model.

To simulate diffusion models, the Euler-Maruyama method might be easy to implement, but has severe short-comings. As it samples whole particle trajectories, it is generally very slow. Furthermore, due to sampling a continuous-time process in discrete time steps, it ignores possible temporary trajectory excursions beyond either boundary between two consecutive trajectory samples (Fig. 1(b)). At each step, this causes it to underestimate the probability of reaching either boundary, and as a consequence, it over-estimates the first-passage times [e.g.^13^]. This leads to conflicting requirements on the simulation step-size. On one hand, it should be small to reduce the first-passage time biases. On the other hand, it should be large to speed up the stimulations. This short-coming also exists in higher-order particle trajectory simulation methods^12^, albeit to a lesser degree.

Here, we introduce a new method to sample from the first-passage time density of simple diffusion models that does not share the short-comings of the Euler-Maruyama and related methods. It does not suffer from any biases, as it directly draws exact samples by rejection sampling from the series expansion of the first-passage time density. Furthermore, it is in the order of a hundred to a thousand times faster than simulating the whole particle trajectory with typical step-sizes. Admittedly, the method is more complex than trajectory-based methods, but the author provides implementations in various programming languages on his website. It is already long know that stochastic simulations that use first-passage time solutions can be more efficient than regular-time-step solution methods. They have, for example, already been used for several decades to simulate non-linear chemical reactions^14^,^15^,^16^. In the context of Wiener diffusion models, a similar approach has been used previously to draw samples from a diffusion model without drift^17^,^18^. The addition of a drift required various changes to the method, that we will discuss in more detail further below. A previously developed sampling method for non-zero drifts^19^ uses a comparable approach to the one described here, but features various weaknesses (see Discussion) that makes it slower than the method presented here.

As described here, the sampling method only applies to diffusion models with symmetric boundaries around a central particle starting point, and a drift and diffusion variance that remains constant over time. However, it is easily embedded in simulations in which these parameters vary across trials. For example, we can draw samples from a model with a drift that varies across trials [e.g.^1^] by first drawing this drift and then using our method to sample the first-passage time and boundary for this drift. The same approach makes it applicable to variable boundaries and non-decision times [e.g.^1^] or the simulation of occasional random lapses^20^. The only restriction is that the boundaries need to remain symmetric around the particle starting point, which makes it unsuitable for situations in which the particle starting point varies [e.g.^1^]. However, even in such cases, our method can be embedded in an approach that provides samples for diffusion models with asymmetric boundaries^19^.

In what follows, we first derive the method based on rejection sampling from the series expansion of the first-passage time density. This includes describing two variants of this series and their relevant properties, the rejection sampling approach, and the derivation of suitable proposal densities for rejection sampling. Then, we perform simulations to evaluate the best settings of the two free parameters of this method, and demonstrate its superiority when compared to the Euler-Maruyama method. Finally, we relate it to previous work and discuss potential extensions.

## Fast and accurate sampling of first-passage time densities

**Figure 1:**
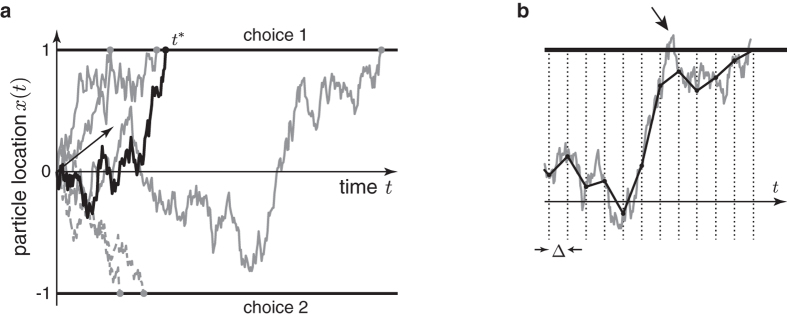
A Wiener diffusion model. (**a**) A diffusion model bounded at 1 and −1, with a positive drift (black arrow extending from origin). A choice is triggered as soon as the drifting and diffusion particle, *x*(*t*), reaches one of the two boundaries. The black example trajectory triggers choice 1 at time t^*^. The stochastic diffusion causes variability in the first-passage times (solid grey trajectories) and choices (dashed grey trajectories). (**b**) Bias of the Euler-Maruyama method applied to simulating diffusion models, due to discretizing a continuous-time process. This method simulates the stochastic process in discrete time-steps of Δ. The discretized trajectory (black line) might miss temporary excursion of the continuous-time trajectory (grey line) beyond the boundary (black arrow), which causes a bias towards higher first-passage times.

We consider a diffusion model in which the trajectory of a drifting and diffusing particle *x*(*t*) is described by the diffusion equation


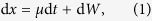


where *μ* denotes the drift and d*W* is a Wiener process (Fig. 1(a)). Initially, the particle is located at *x*(0) = 0. It terminates its trajectory at some time *t* > 0 as soon as it reaches one of two boundaries, located at −1 and 1. The time at which either boundary is reached is known as the *first-passage time*. In the following, we describe a fast method to draw samples from both first-passage time and boundary. After that, we describe how the same procedure can be used to draw samples from diffusion models with non-unit boundary locations and non-unit diffusion variance.

### First-passage time densities

For the considered diffusion process, the first-passage time densities *g*_*s*,+_(*t*) for the upper boundary, and *g*_*s*,−_(*t*) for the lower boundary can be expressed as an infinite series. The series that will be described are both exact in the infinite limit, but converge to the correct density at different rates, depending on *t*. The first series is found by the method of images^21^, resulting in


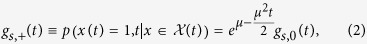



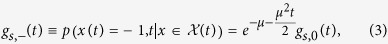


where 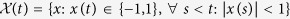 is the set of all particle trajectories that reach either of the boundaries for the first time at time *t*. In the above, *g*_*s*,0_(*t*) denotes the infinite series


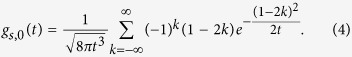


This series is known to converge rapidly for small times^10^,^17^,^18^, which is indicated by its subscript 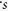 for *short-time* series.

An alternative approach to compute the first-passage time densities is to seek a Fourier series solution^21^, which, after some simplification due to symmetric boundaries, results in


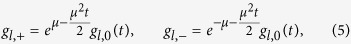


with the corresponding infinite series


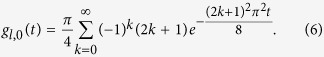


This series converges rapidly for large times^10^,^17^,^18^, which is indicated by its subscript 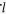 for long-time series.

A useful property of diffusion models with symmetric boundaries is that their first-passage times are independent of which boundary is reached. To see this, consider Eqs. (2) and (3), which reveal that these joint probabilities are re-scaled versions of each other, whose scaling depends only on the drift *μ*, but not on time *t*. As a result, they can be factored into


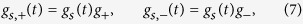


where *g*_*s*_(*t*) is the first-passage time irrespective of which boundary was reached,


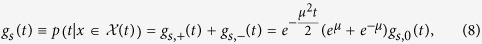


and *g*_+_ and *g*_−_ are the probabilities of reaching the upper and lower boundary, respectively, irrespective of the first-passage time,


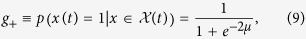



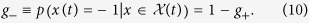


The same follows from Eq. (5) for the long-time version, with the corresponding first-passage time density 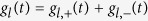.

A consequence of this factorization is that the first-passage time can be sampled independently of which boundary was reached. Furthermore, sampling the boundary corresponds to a draw from a Bernoulli distribution with probability *g*_+_. Thus, the rest of this section focuses on how to draw samples from the first-passage time density, using either *g*_*s*_(*t*) or *g*_*l*_(*t*).

### Rejection sampling from a series expansion

To sample from the first-passage time density, we have to overcome several obstacles. First, it is not possible to draw samples from this density directly. We tackle this problem by rejection sampling, for which it is sufficient to find an easy-to-sample proposal density that upper-bounds the first-passage time target density up to a proportionality constant. Second, such rejection sampling requires the accurate evaluation of the value of this target density for particular times. However, the density is only available as a series expansion, which prohibits such accurate computation, and makes even approximate evaluations computationally expensive. Fortunately, both described series expansions feature a property that makes them suitable for a variant of rejection sampling that only requires the computation of few elements in these series, such that exact samples can be drawn without the use of approximations. Third, each of the two series only features this property for a limited range of first-passage times. In combination, they cover all non-negative times, such that, depending on the time, one or the other can be chosen. However, the series feature qualitatively different properties that need to be taken into account when designing suitable proposal densities for rejection sampling. In the below, we handle each of these points in turn.

**Figure 2:**
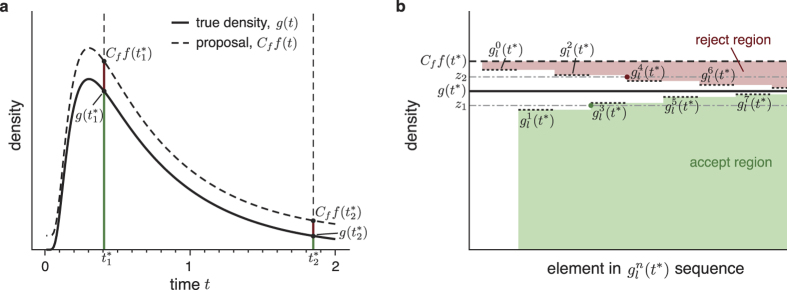
Rejection sampling from a convergent series. (**a**) shows the true first-passage target density *g*(*t*) for a drift of *μ* = 1, and some proposal density *f*(*t*) that upper-bounds this target density up to some proportionality constant *C*_*f*_. Assume drawing 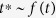 and 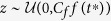. Then rejection sampling accepts t^*^ if and only if 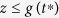. It is important that 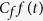 tightly upper-bounds *g*(*t*), as illustrated by two samples, 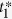 and 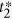. Either sample is accepted if the corresponding *z* falls into the green region, and rejected if it falls into the red region. Thus, the first sample, 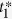 is very likely accepted, while 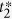 is more likely to be rejected, due to 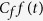 only loosely bounding *g*(*t*) in the tails. (**b**) The sequence 
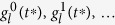
 converges to 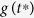 from above and below, thus forming a reject/accept region around 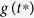 that increases in size with every additional element of this sequence. Thus, the t^*^ can be accepted (for *z*_1_, green dot) or rejected (for *z*_2_, red dot) while computing only a small number of these elements.

Rejection sampling is a sampling method in which one draws a sample t^*^ from an easy-to-sample proposal density 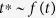 that tightly upper-bounds the desired sampling density *g*(*t*), up to a proportionality constant *C*_*f*_ (Fig. 2(a)). That is 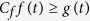 for all *t*. Assuming for now that 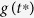 can be computed rapidly and accurately, this sample is accepted if 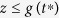, where 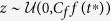 is a sample from the uniform distribution over 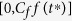. Otherwise, sampling is repeated until the first sample is accepted [22, Ch. 2].

The efficiency of rejection sampling hinges on how likely the samples drawn from the proposal density are accepted. To ensure a high acceptance likelihood, the scaled proposal density 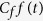 needs to tightly upper-bound the target density *g*(*t*), as illustrated in Fig. 2(a). This is particularly important for the tails of the target density. A proposal density that only loosely bounds these tails causes more samples to be drawn from these tail regions which are in turn very likely rejected (e.g. sample 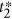 in Fig. 2(a)). Thus, great care will be put on tightly bounding the tail regions when designing suitable proposal densities.

We cannot directly apply rejection sampling to draw samples from the first-passage times, as *g*(t^*^) is only known in the form of infinite series. However, these series have a property that makes them suitable for the series method, which is a particular variant of rejection sampling [22, Ch. 4]. Focusing for now on the long-time series used in 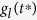, we will show in the next section that its elements form a sequence that alternatingly upper and lower-bound the true 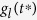 (Fig. 2(b)). That is, for sufficiently large t^*^ we can form a easy-to-compute sequence 
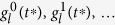
 for which 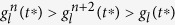 for all even *n*’s, and 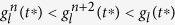 for all odd *n*’s. As a result, we can define a region 
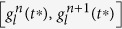
 (assuming odd *n*) that is guaranteed to contain 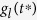, and whose size shrinks towards zero for increasing *n*.

With such a sequence, rejection sampling can be performed as illustrated in Fig. 2(b). First, we again draw a sample t^*^ from some proposal density *f*(*t*) that upper-bounds 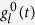, and for which 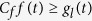 for all *t*. Additionally, we draw 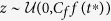. If 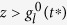, the sample of t^*^ is immediately rejected, and if 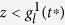 it is immediately accepted. Otherwise, we proceed iteratively to narrow the region around 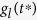 until rejection or acceptance occurs. For *n* = 2 and any subsequent even *n*, we compute 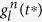 and reject t^*^ if 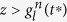. Otherwise, we increment *n* by one, compute 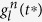, and accept t^*^ if 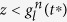. If neither occurred, we increment *n* by one and return to the previous step for even *n*’s. This procedure is repeated until t^*^ is either accepted or rejected. As the sequence of 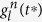
’s converges to 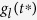, the procedure is guaranteed to terminate.

In the following we show that such a convergent series can be found for both *g*_*s*_(*t*) and *g*_*l*_(*t*). This convergence is only guaranteed for small *t* for *g*_*s*_(*t*) and large *t* for *g*_*l*_(*t*), such that the choice of series depends on the initially sampled t^*^. After having shown this, we design suitable proposal densities for both *g*_*s*_(*t*) and *g*_*l*_(*t*).

### Sequences of upper and lower bounds on *g*_*s*_(*t*) and *g*_*l*_(*t*)

Considering first the short-time series, we show how to construct a sequence 
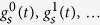
 that converges to *g*_*s*_(*t*) in Eq. (8) from above and below for even and odd *n*’s, respectively. For any fixed time *t*, *g*_*s*_(*t*) is a scaled version of 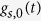, Eq. (4), such that it is sufficient to construct such a sequence on 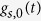. Before doing so, note that the *k*th term of 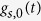 is equivalent to the (1 − *k*)th term, such that they can be re-grouped into a more convenient form, given by


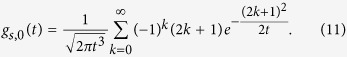


Based on this, we define the truncated series


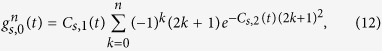


with 
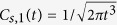
 and 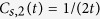. This series can be computed recursively by


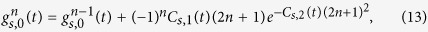


starting with 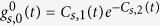. Using the above recursion, it is easy to show that 
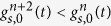
 for even *n*, and 
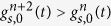
 for odd *n*, as long as


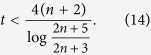


holds. Overall, to satisfy the above for all *n*, we need to have


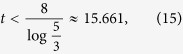


thus limiting the use of the short-time series to these values of *t*. Within this range, 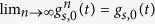 implies that 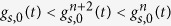 for even *n* and 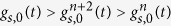 for odd *n*, as required by our sampling method.

For the long-time series, we define


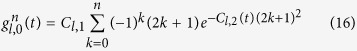


with 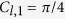 and 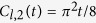, as the truncated series approaching 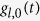, Eq. (6). As for the short-time series, this truncated series can be computed recursively by


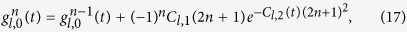


starting with 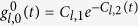. Using the same procedure as before, it is easy to show that, as long as


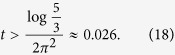


we can guarantee that 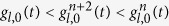 for even *n* and 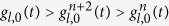 for odd *n*, as required by our sampling method.

Overall, the two series differ only in their applicable range of *t* and in the constants that they rely on. Therefore, as shown in Table 1, the accept/reject decision for exact sampling with both series can be implemented by the same function that takes the series-dependent *C*_2_ as an argument.

### Suitable proposal distributions

What remains is to define a proposal density to draw the t^*^’s from. This proposal *f*(*t*) needs to be easy to sample from, and, scaled by *C*_*f*_, needs to tightly upper-bound the density we wish to sample from. As previously discussed, the tightness of this bound is important, as it determines the rejection rate, and thus the efficiency of the sampling procedure. Here, we construct two such proposal densities. The first, *f*_1_(*t*), is tight for small *μ*, and the second, *f*_2_(*t*), for large *μ*. The final sampling scheme chooses between these proposal densities on the basis of comparing the desired *μ* to some threshold 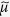. In addition to this, we assume some 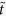 below and above which the short-time and long-time series are used, respectively, to decide if the drawn t^*^ is accepted. For now, we only require 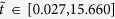 (from Eqs. (15) and (18)), but later we will tune this parameter to maximize sampling performance.

**Figure 3:**
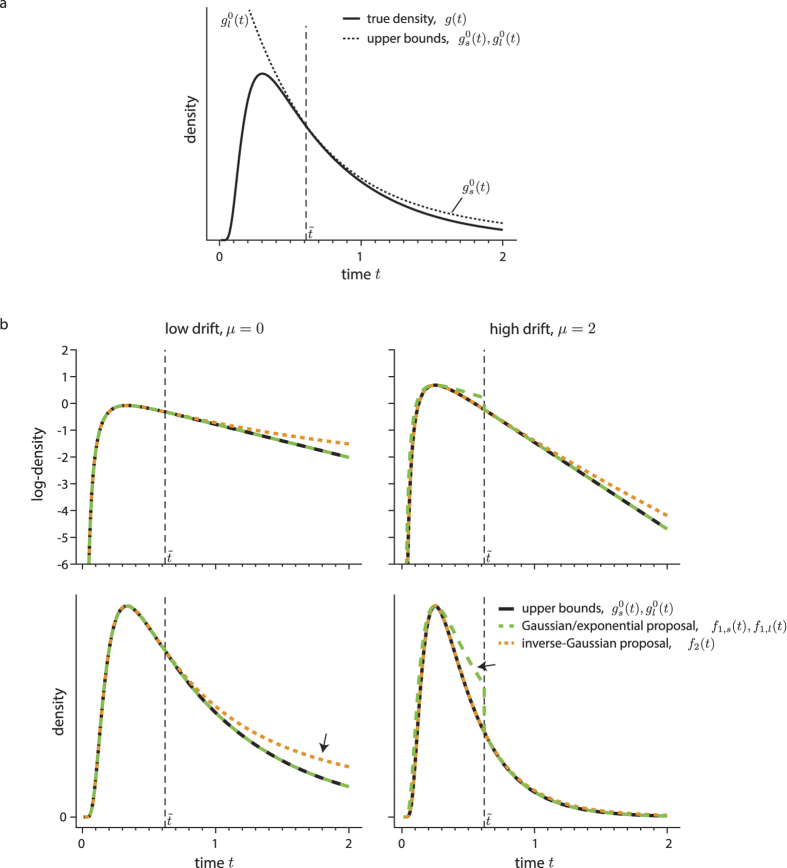
The upper bounds on the first-passage time, and proposal densities for different drifts *μ*. (**a**) shows the true first-passage time density *g*(*t*), and the two upper bounds, 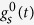 and 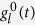, resulting from a truncation of the respective series expansion. For low *t*, 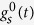 is indistinguishable from *g*(*t*). The same applies to 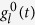 for large *t*. (**b**) shows the proposal density for low and high drift *μ*. As can be seen, the Gaussian/exponential proposal, 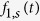 and 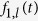, tightly upper-bounds 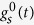 and 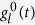 for small *μ* (right panels). For such *μ*, the inverse-Gaussian proposal, 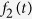, is less tight for larger *t* (black arrow, lower right panel). For larger *μ*, 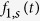 only loosely upper-bounds 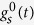 (black arrow, lower left panel). In these cases, 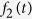 becomes a better proposal density. For illustration, the threshold 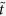 between the short and long-time series was in all panels fixed to 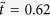.

To use the series-based accept/reject procedure, either proposal density should upper-bound the largest bound in the sequence that approaches 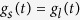. For both the short-time and long-time series, this bound is given by the *n* = 0 element in the respective sequence, such that, by combining Eqs. (8), (12) and (16), these bounds are given by


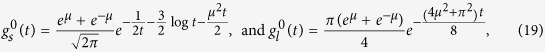


as illustrated in Fig. 3(a).

#### Proposal distribution for small *μ*

In the above, 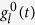 is proportional to the density of an exponential distribution with rate 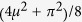. This distribution is easy to sample from, such that we choose


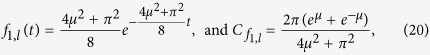


where 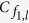 was chosen for 
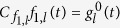
 to hold.

For small *μ*, 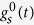 turns out to be tightly bounded by


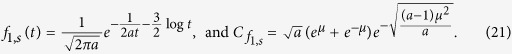


for some *a* ≥ 1. The proposal 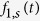 can be shown to emerge from 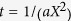, where *X* is a standard Gaussian random variable with zero mean and unit variance. 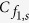 was chosen to have 
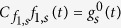
 for at least one *t*, and 
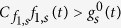
 otherwise. We choose the *t* at which the two functions touch to be at the mode of 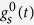, resulting in


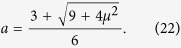


To sample from this proposal, we cannot directly use Gaussian samples, as these would only be useful for the short-time part of the support of the proposal density. Instead, we use the inversion method [22, Ch. 2], which first draws a value from a uniform distribution and then transforms this value by the inverse cumulative function to achieve samples from the desired density. Thus, the method requires the full cumulative functions of the proposal densities. For 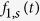, this cumulative function and its inverse are given by


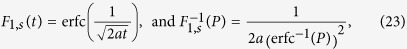


where 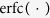 and 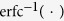 are the complementary error function and its inverse, respectively. For 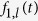, we find


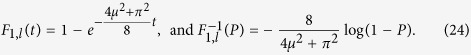


Combining 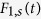 and 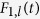 at 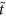, and adding the appropriate scaling constants results in the overall cumulative


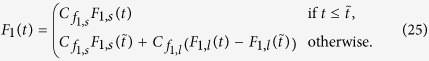


This cumulative function has limit 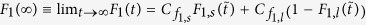. The densities that the cumulative functions are based on are unnormalized, such that 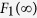 is not guaranteed to be one, as would be the case for normalized densities. Thus, to sample according to *F*_1_(*t*), we draw a *P* uniformly from 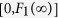 (rather than from 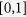, as we would for normalized densities), and then choose t^*^ according to


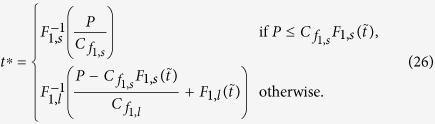


Overall, this results in the algorithm in Table 2, where we have used


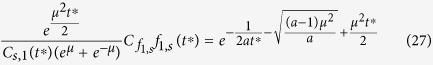


for the adequately re-scaled short-time proposal at t^*^, and the corresponding


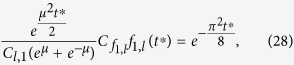


for the long-time proposal.

#### Proposal distribution for large *μ*

As Fig. 3(b) illustrates, the described proposal forms a tight upper bound on 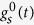 for small *μ*, but fails to do so for large *μ*. In case of the latter, we use the property that 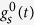 is proportional to an inverse-Gaussian distribution with mean 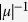 and shape 1, which we can sample from using the proposal density


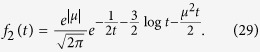


A scaling constant of 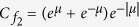 ensures that 
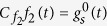
, but it is not guaranteed to upper-bound 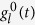 for all 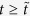. 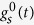 and 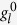 intersect at 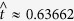, above which 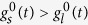 is guaranteed (see Fig. 3(a)). Thus, as long as 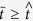, the above 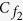 causes the proposal to upper-bound 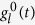. As soon as 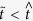, we need to additionally re-scale the proposal by


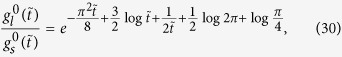


leading to the final scaling constant


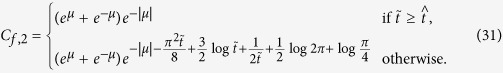


Figure 3(b) shows that this proposal provides a tighter bound for larger *μ*. Overall, this results in the algorithm in Table 3, whose constants *C*_*s*_ and *C*_*l*_ are based on an adequate re-scaling of the proposal density, and where 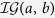 denotes an inverse-Gaussian distribution with mean *a* and scale *b*.

### The complete method

As Fig. 3(b) illustrates, the Gaussian/exponential proposal, 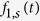 and 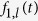, is tight for small *μ*, and the inverse-Gaussian proposal 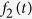 is tight for large *μ*. Therefore, we choose between them according to some threshold 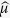, to be determined later.

To sample from more general diffusion models with drift 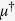, non-unit variance *σ*^2^ and boundaries at *θ* and −*θ*, we re-scale particle location *x* and time *t* in Eq. (1) by 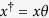 and 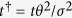, resulting in


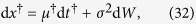


where *μ* in the base diffusion model relates to 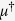 by 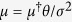. Once *x* in the base diffusion model reaches −1 or 1, 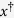 in the re-scaled model reaches −*θ* or *θ*. Therefore, we can sample from the more general model by drawing a sample t^*^ from the base diffusion model with drift *μ*, and then re-scaling this sample according to 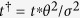.

This leads to the algorithm in Table 4, which returns a sample of the first-passage time, together with which boundary was reached first. In this algorithm, 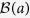 denotes a draw from a Bernoulli distribution with probability *a*, where we have used the boundary probability *g*_+_ as given by Eq. (9).

## Simulations

In this section we show by simulations how to set the thresholds 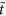 and 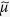, and demonstrate the speed-up the the proposed method achieves when compared to simulating a diffusion model by the Euler-Maruyama method. All reported sampling speeds result from a single-core Julia implementation of the algorithms, running on a Mid-2014 15” MacBook Pro with a 2.8 GHz Intel Core i7 processor and 16 GB of RAM. Implementations of the proposed algorithm are available on the author’s webpage in Julia, as well as in C++11, with MATLAB and Python interfaces.

### Tuning the thresholds 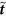 and 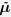


**Figure 4:**
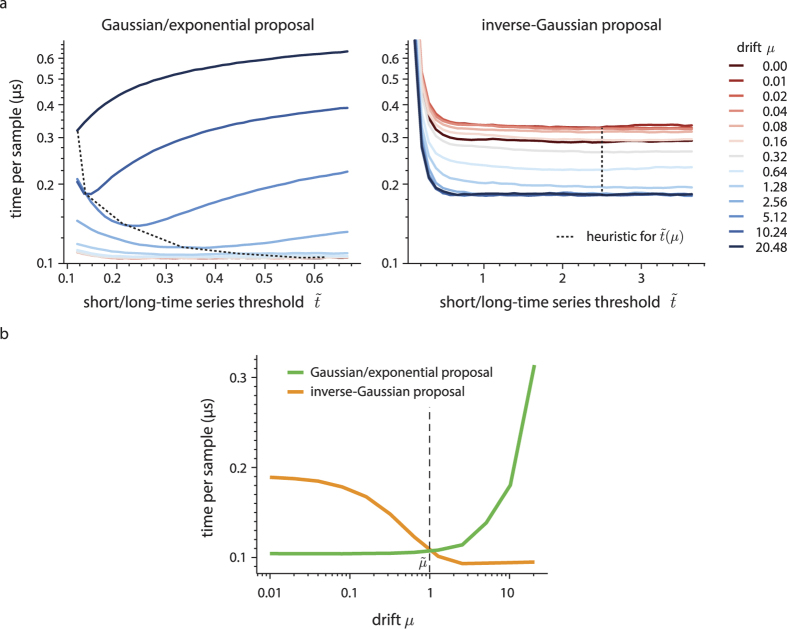
Average time per sample as function of the drift *μ* and threshold 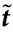. The threshold 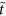 determines if accept/reject is determined by the short-time or long-time series. (**a**) shows the sampling speed for both the Gaussian/exponential and the inverse-Gaussian proposal for different drifts and thresholds. The black, dashed line corresponds to 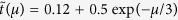 and 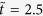 for the Gaussian/exponential and inverse-Gaussian proposals, respectively. It shows the sample speeds associated with the chosen threshold values. (**b**) shows how the sampling speeds for the two proposals with tuned thresholds 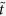 depends on the drift *μ*, and the resulting threshold 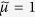 that determines which proposal to use. The sampling times are generally lower than in (**a**), as fixing 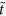 leads in some cases to a simplification of the sampling algorithm.

We start by finding the best threshold 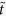 between the short-time and long-time series expansion for both proposal distributions. In all cases, sampling speed is on one hand determined by how tight the proposal upper-bounds 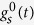 and 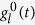, and, on the other hand, by how many elements in the corresponding sequence need to be evaluated before the proposed t^*^ sample can be accepted or rejected. Also, a change in drift *μ* causes a change in both the target density and the proposals. To take all of these factors into account, we measured the time it took to draw 10^6^ samples for a set of different 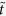 and *μ*. For each 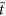 and *μ* we repeated this procedure 100 times, discarded the top and bottom 20% sampling times as outliers, and averaged over the rest. The resulting average sampling times are shown in Fig. 4(a).

For the Gaussian/exponential proposal, 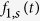 and 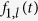, Fig. 4(a) shows that, for small *μ*, sampling speed is mostly independent of the choice of 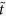, as long as 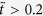. As soon as *μ* rises above around 1, increasingly smaller 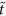 lead to more rapid sampling. The relationship between *μ* and the 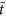 that maximizes sampling speed turned out to be well captured by 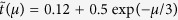 (dashed line in Fig. 4), which ranges from 0.62 for small *μ* to 0.12 for large *μ*. We acquired this function to set 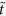 for all future uses of this proposal.

For the inverse-Gaussian proposal, 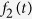, Fig. 4(a) demonstrates that *μ* and 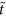 influence the sampling speed largely independently. An increasing drift *μ* generally causes faster sampling, which can be traced back to 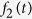 being a tighter upper bound in such cases. The threshold 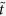 did not influence the proposal 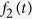 directly, but modulated sampling speed by determining which series was used to accept or reject the drawn time samples. Sampling was slow for small 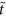, but for 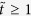, this threshold had little influence on the sampling speed. Thus, independent of the drift, we chose 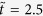 for this proposal (dashed line in Fig. 4), which gave the overall best performance.

Having determined a tuned 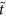 for each proposal, we now turn to the question of how to set 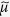 to choose between the two proposal densities. To do so, we evaluated the sampling speed associated with either proposal as before, for a set of different *μ*, but this time using the tuned 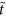. In particular for the inverse-Gaussian proposal, using this 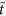 led to simplified algorithms (see Table 3 for 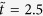), and an associated increase in sampling speed. The resulting speeds for both proposals are shown in Fig. 4(b). As expected, the Gaussian/exponential proposal performs better for small *μ*, and the inverse-Gaussian proposal for large *μ*. Their speeds intersect at around 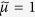 (dashed line in Fig. 4(b)), which we acquired as the threshold to decide between the two proposal densities.

### Speed-up when compared to the Euler-Maruyama method

**Figure 5:**
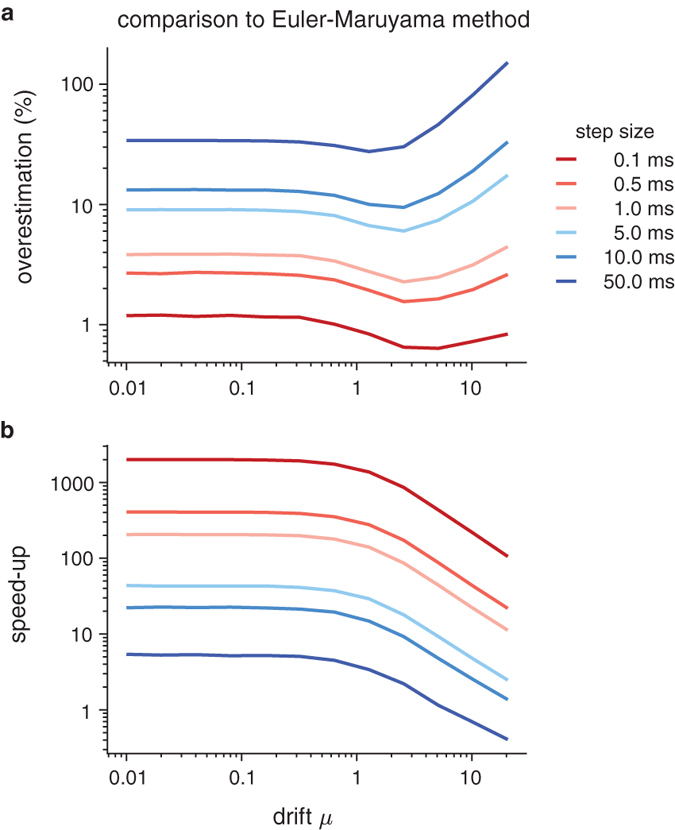
Comparison of our method to the Euler-Maruyama method. (**a**) shows the overestimation of first-passage time resulting from the Euler-Maruyama method for different step-sizes Δ and drifts *μ*. For each Δ and *μ* we computed the average first-passage time by averaging over 10^7^ Euler-Maruyama simulations of a diffusion model. The degree of over-estimation was found by dividing this average by the analytical expression for this average, given by tanh(*μ*)/*μ* if *μ* > 0 and 1 otherwise^21^. (**b**) illustrates the speed-up achieved by our method, when compared to the Euler-Maruyama method, for different step-size Δ for the latter, and different drifts *μ*. A speed-up of 10 means that, on average, our method yields samples ten times faster than the Euler-Maruyama method.

To get an idea of the speed-up achieved by the proposed method, we compare it to the standard Euler-Maruyama method for simulating diffusion models. This method starts at *x*^0^ = 0 and then iterates over


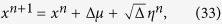


until 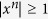. In the above, Δ is a small step-size, and *η*^*n*^ is a zero-mean unit-variance Gaussian random variable. While easy to implement, the algorithm does not take into account excursions of the *x*(*t*) trajectory beyond −1 or 1 between two consecutive trajectory samples, *x*^*n*^ and *x*^*n*+1^ (see Fig. 1(b)), which makes it prone to over-estimating the first-passage time^13^. The resulting bias is shown in Fig. 5(a) for different step-sizes Δ and drifts *μ*, which illustrates that larger step-sizes cause an increase in the bias. Taking larger steps also lowers simulation time, such that the choice of Δ is a trade-off between minimizing bias and maximizing sampling speed. This trade-off is not present in our method, which always generates unbiased first-passage time samples.

As there is no single best step-size for the Euler-Maruyama method, we compared the speed of our method to that of the Euler-Maruyama method for different step-sizes. For either method, we found the sampling speed as before, by computing an average over 100 runs of 10^6^ samples each, while discarding the slowest and fastest 20% of these runs as outliers. As shown in Fig. 5(b), this procedure revealed a speed-up by a factor of 100 to 1000 for sensible step-sizes, Δ ≤ 1 *ms*, as recommended by (ref. 12). Even for extreme step-sizes of Δ = 50 *ms*, in which the Euler-Maruyama method might over-estimate the first-passage times by a factor of two, our method featured faster sampling times for *μ* < 5. Thus, there does not appear any sensible parameter range in which the Euler-Maruyama method yielded lower sampling times than our method. For this reason, our method should always be the preferred approach.

## Discussion

We have developed a fast and unbiased method to sample first-passage times from diffusion models. This method is based on rejection sampling from the known infinite series expansion to the first-passage time densities. Making use of properties of this series, we showed that samples can be rejected or accepted while computing only few terms of this series. The method features two parameters that we have tuned by simulations to maximize sampling speed. Overall, our method draws unbiased samples roughly a hundred to a thousand times faster than the Euler-Maruyama method that only provides biased samples.

Previously, a similar approach has been used to draw samples from a diffusion model with zero drift^17^,^18^. This allowed the authors to directly use the upper bounds, 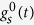 and 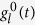, as proposal densities, and draw samples from the resulting density by the inverse method. Once we introduced a non-zero drift, these densities became inadequate, such that we had to replace that bounding 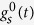 with a time-rescaled variant, 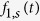. Even then, the proposal only loosely bounds 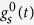 for large *μ*, which might lead to a large rejection rate and thus inefficient sampling. For this reason, we used another inverse-Gaussian proposal density 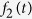 that is tighter for *μ* > 1 and, as a consequence, provides faster sampling for such *μ*. Interestingly, this proposal corresponds to the first-passage time density for diffusion models with a single bound^21^. Hence, as soon as the drift towards this bound is sufficiently large, the contribution of the opposing bound to this density becomes negligible.

A previously proposed approach^19^ for non-zero drifts is also based on rejection sampling and so comparable to the method proposed here. It differs from our method in the following points. First, it features a single, less-tight proposal density whose acceptance rate decreases with increasing drift rates. Our method avoids this by using different proposal densities for small and large drift rates. Second, the previous approach does not use the alternating lower/upper-bound property of the series expansion of the first-passage time densities to guide rejection, but instead truncates this expansion after a fixed number of terms. If the expansion is truncated after too few terms, sampling will be inaccurate. If too many terms are evaluated, the method will be slower than ours. Third, the series expansion used in^19^ corresponds to the Fourier series solution, Eq. (5) which is known to converge quickly for large drift rates, but slowly for small drift rates. Therefore, the number of terms after which to best truncate Eq. (5) depends on the drift rate, which is not considered in (ref. 19). We, instead, use a different series for small drift rates, which makes the method overall faster.

In terms of sampling speed, we compared our method to the Euler-Maruyama method, which is known to be both biased and slow. Higher-order alternatives to the stimulation of stochastic differential equations [e.g.^23^] might reduce this bias, and thus might seem a more adequate performance baseline. However, their lower bias comes at a higher computational cost, which makes them slower than the Euler-Maruyama method. Also, while they might be able to lower the bias, they will not be able to completely eliminate it. Therefore, even these higher-order methods will be no match to the method we have proposed. Furthermore, for the sake of fitting diffusion model parameters, they only provide a marginal improvement over the most basic Euler-Maruyama method^12^.

While our method was developed for a simple diffusion model with symmetric unit boundaries and a unit diffusion variance, time and space rescaling makes it applicable to arbitrary boundary levels and diffusion variances. Furthermore, it can be embedded within a sampler that also models drifts, bounds, and other variables as random, thus providing additional levels of flexibility [e.g.^1^,^11^]. One restriction for our method to work is that the boundaries need to be symmetric around the particle starting point. This restriction ensures that both the short-time and long-time series alternatingly form upper and lower bounds on the true first-passage time density. For asymmetric boundaries, this property is not guaranteed for the long-time series, such that we are unable to use the same rejection sampling variant.

**Figure 6:**
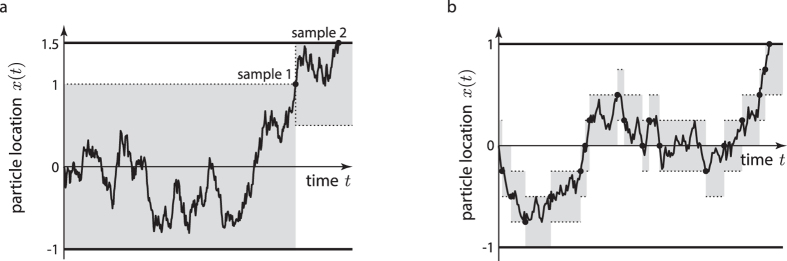
Possible extensions of the proposed method. (**a**) Application to diffusion models with asymmetric boundaries. Such boundaries can be handled by using a symmetric diffusion model centered on the current particle location that is tightly bounded by the asymmetric diffusion model (shaded areas). A sample is returned if a boundary shared by both diffusion models is reached (sample 2). Otherwise (sample 1), a new symmetric diffusion model is inscribed, and the procedure is repeated. (**b**) Approximating a leaky accumulator. A leaky accumulator is governed by the stochastic process 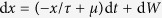, but can be locally approximated by 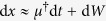, where 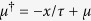. Splitting *x* into equally-sized regions, each endowed with a different drift 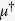, results in the piece-wise sampling scheme illustrated above.

One possible extension to sample efficiently from diffusion models with asymmetric boundaries is illustrated in Fig. 6(a). The idea is to use the symmetric sampler as a building block to sample from more complex diffusion models, analogous to the method introduced by (refs. 17, 18, 19). In the case of asymmetric boundaries, sampling would commence by assuming a symmetric diffusion model that is tightly bounded by the asymmetric model. When reaching the boundary that is shared by both models, sampling would stop and return the sampled time and boundary. Otherwise, sampling continues from another symmetric diffusion model that is again tightly bounded by the asymmetric model, but is this time centered on the previously terminal particle location. This procedure is continued until the reached boundary is that shared by both the symmetric and the asymmetric model. At that point, the total time, as well as the reached boundary are returned.

A similar approach allows us to approximate samples from an Ornstein-Uhlenbeck process, or leaky accumulator, which acts as another popular psychological model^24^. In this case, we could approximate the leak, which theoretically varies continuously over the particle space, by a sequence of regionally constrained leak-free diffusions, each of which are represented by symmetric diffusion models with a different drift (Fig. 6(b)). Due to the approximation, this approach would unfortunately introduce a bias. An unbiased alternative is to use a method that samples from such leaky processes without bias, by creating a sequence of skeleton point connected by Brownian bridges^25^. While this has the potential for faithfully sampling from leaky processes, its efficiency when compared to the the Euler-Maruyama method remains to be evaluated.

## Conclusions

We have presented a new method to sample the first-passage time and reached boundary for Wiener diffusion models. Our method is superior to previously used approaches in that it is both unbiased and significantly faster. While restricted to diffusion models with boundaries symmetric around the starting point, it can act as a building block to sample from models that violate this constraint. Thus, it promises to extend its reach, improving upon both fitting such models to behavioral data and simulating them with high efficiency.

## Additional Information

**How to cite this article**: Drugowitsch, J. Fast and accurate Monte Carlo sampling of first-passage times from Wiener diffusion models. *Sci. Rep.* **6**, 20490; doi: 10.1038/srep20490 (2016).

## Figures and Tables

**Table 1:**
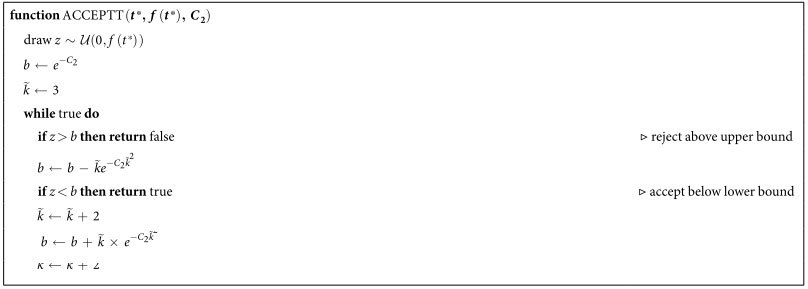
Accept/reject algorithm for converging series.

t^*^ is a sample drawn according to proposal *f*(*t*), where *f*(*t*) strictly dominates 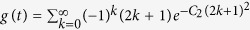. The function returns if t^*^ is to be accepted/rejected.

**Table 2:**
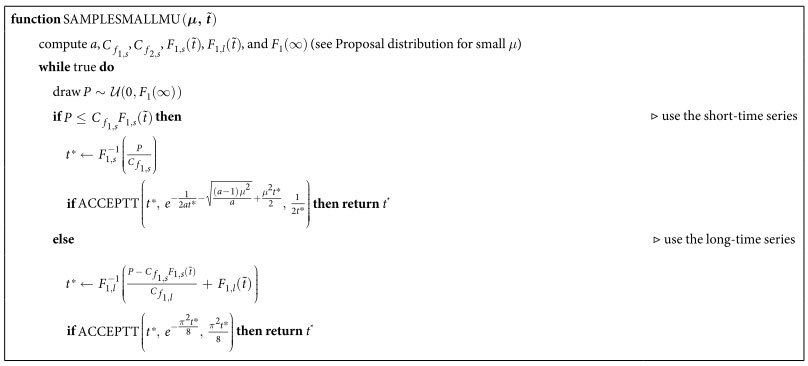
Algorithm to sample first-passage times for small *μ*.

**Table 3:**
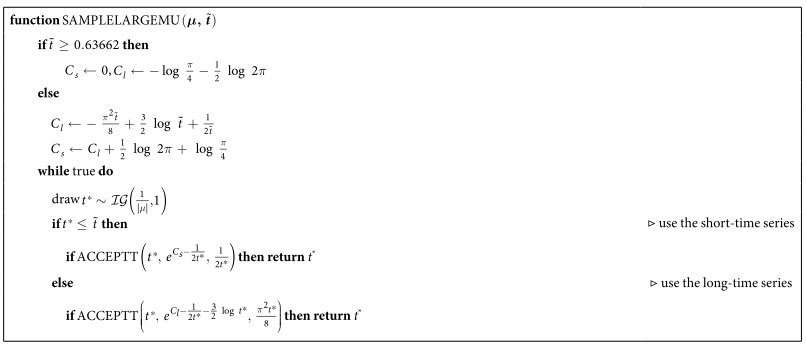
Algorithm to sample first-passage times for large *μ*.

**Table 4:**
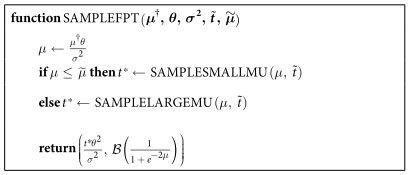
The complete sampling algorithm.

The function takes the drift 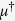, boundary *θ*, and diffusion variance *σ*^2^ and returns the tuple (*T*, *X*), where *T* is a sample of the first-passage time, and *X* = 1 (*X* = 0) if the upper (lower) boundary was reached first. 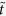 and 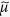 are tuning parameters whose values are optimized in the Simulations section.
